# New Directions for SMA Therapy

**DOI:** 10.3390/jcm7090251

**Published:** 2018-08-31

**Authors:** Sonia Messina

**Affiliations:** 1 Department of Clinical and Experimental Medicine, University of Messina, 98100 Messina, Italy; smessina@unime.it; Tel.: +39-090-2212793; 2 NEuroMuscular Omnicentre (NEMO) Sud Clinical Centre, University Hospital “G. Martino”, 98125 Messina, Italy

**Keywords:** antisense oligonucleotides, gene therapy, spinal muscular atrophy, therapy, nusinersen

## Abstract

Spinal muscular atrophy (SMA) is a severe disorder of motor neurons and the most frequent genetic cause of mortality in childhood, due to respiratory complications. The disease occurs due to mutations in the survival motor neuron 1 (SMN1) gene that leads to a reduction in the SMN protein, causing degeneration of lower motor neurons, muscle weakness and atrophy. Recently, the Food and Drug Administration (FDA) and the European Medical Agency (EMA) approved the antisense oligonucleotide nusinersen, the first disease-modifying treatment for SMA. Encouraging results from SMN1 gene therapy studies have raised hope for other therapeutic approaches that might arise in the coming years. However, nusinersen licensing has created ethical, medical, and financial implications that will need to be addressed. In this review, the history and challenges of the new SMA therapeutic strategies are highlighted.

## 1. Introduction

Spinal muscular atrophy (SMA) is a heterogeneous hereditary neuromuscular disease, presenting with progressive weakness of skeletal and respiratory muscles, leading to muscle atrophy. The clinical subtypes are based on the age of onset and the maximum motor milestones achieved, ranging from the most severe SMA I to the mildest SMA IV. SMA I patients never acquire the sitting position and usually die of respiratory muscle failure within the first years of life [1]. SMA II patients are able to sit but not walk independently, and are characterized by orthopedic complications such as joint contractures, severe scoliosis, and respiratory involvement that usually requires the use of non-invasive ventilation before adulthood. SMA III and IV are the mildest forms, in which patients are able to walk independently and have a variable clinical course, usually without life-threatening events before adulthood. SMA I and II patients often require nutritional support with the use of hypercaloric and hyperproteic oral supplements, or more invasive interventions such as gastrostomy. These patients require a multidisciplinary team to provide the necessary respiratory, orthopedic and nutritional care. SMA is one of the most common autosomal recessive diseases and causes of mortality in childhood, with an incidence of 1 in 11,000 live births and a carrier frequency of 1 in 40–67 adults [2]. This disease is caused by mutations in the survival motor neuron 1 (SMN1) gene and consequential lack of the SMN proteins [3].

Because of its central role in the pathogenesis of SMA, SMN’s cellular role has been extensively studied. SMN is a ubiquitously expressed 32-kDa protein. SMN has an important housekeeping role in all cells with regard to regulating the biogenesis of ribonucleoprotein (RNP) complexes. SMN has, in fact, a general role in RNP formation, as illustrated by defects in the formation when SMN levels are low, of small nucleolar RNPs (snoRNPs) required for the post-translational modification of non-coding RNAs, messenger RNPs (mRNPs) involved in the transport of mRNAs, and signal recognition particles (SRPs) which regulate the transport of newly synthesized proteins. SMN depletion affects a number of other cellular pathways that are of particular interest for the maintenance of neuronal homeostasis. For example, actin dynamics, ubiquitin homeostasis, mitochondrial function and endocytosis are all disrupted in SMA [3].

SMN2 is an SMN1 paralogous gene, producing a protein lacking exon 7 (SMNΔ7) due to alternative splicing, but also small amounts of functional SMN proteins [4]. The number of SMN2 copies is variable, and because each copy produces low levels of full-length, functional SMN, the number of SMN2 copies is inversely related to symptom severity and clinical subtype [5]. However, both between patients with the same number of SMN2 copies and within families, considerable variation in clinical phenotypes can occur. Indeed, research on discordant families has led to the identification of several genetic modifiers of SMA, including plastin-3 and neurocalcin delta. It is likely, therefore, that further genetic and other disease-modifying factors are still to be discovered.

This review offers an outline of the progress made, and the challenges facing new therapeutic strategies in the treatment of SMA.

## 2. Survival Motor Neuron (SMN)-Dependent Gene Therapies

### 2.1. SMN2 Modulators

#### 2.1.1. Nusinersen

The only therapy that is presently approved for SMA is nusinersen (SPINRAZA), which is an antisense-oligonucleotide (ASO) that binds to the SMN2 pre-mRNA downstream of exon 7, promoting its incorporation into the mRNA and leading to the translation of a fully functional SMN protein [6].

Several trials have demonstrated evidence of nusinersen efficacy without any major adverse effects (Table 1). In all clinical trials, there was an initial loading period in which nusinersen was intrathecally administered four times over two months, followed by a maintenance period with injections every four months.

ENDEAR was a phase III, randomized, double-blind, sham-procedure controlled study (ClinicalTrials.gov identifier: NCT02193074, conducted over the years 2014–2016) to assess the clinical efficacy and safety of nusinersen administered intrathecally in infantile-onset SMA [7]. The primary outcomes were the percentage of motor-milestone responders (based on improvement in the motor milestone categories of the Hammersmith Infant Neurological Examination) and time to death or permanent ventilation. In the interim analysis, infants treated with nusinersen had higher motor-milestone responses than controls (41% vs. 0%, *p* < 0.001) and therefore the trial was stopped, and patients continued treatment in an open-label extension study (SHINE). Moreover, the nusinersen group had longer survival periods and a longer time until they required permanent ventilation compared to controls.

CHERISH was a phase III, randomized, double-blind, sham-procedure controlled study (ClinicalTrials.gov identifier: NCT02292537, conducted over the years 2014–2017) to assess the clinical efficacy and safety of nusinersen administered intrathecally in patients with later-onset SMA [8]. The primary outcome measure was change from baseline in the Hammersmith Functional Motor Scale-Expanded (HFMSE) score after 15 months of treatment. In the interim analysis, the nusinersen group had a mean increase of 4.0 points versus a mean decrease of 1.9 points in controls (*p* < 0.001). For this reason, the trial was ended and patients were included in the aforementioned SHINE study. In the final analysis, 57% of nusinersen patients versus 26% in the sham group had a rise of three points in HFMSE scores after 15 months of treatment.

NURTURE is an open-label study (ClinicalTrials.gov identifier: NCT02386553, started in 2015 and still ongoing) to assess the efficacy, safety, tolerability and pharmacokinetics of multiple intrathecal doses of nusinersen administered to presymptomatic SMA infants younger than six weeks old. The effects of nusinersen were encouraging, with improvement in motor milestones and growth parameters, no fatalities, and no ventilation required after 323 days [9]. The favorable results of this trial support the rationale for SMA newborn screening, so that treatment can be started earlier and greater improvements can be achieved.

The SHINE study (ClinicalTrials.gov identifier: NCT02594124) started in 2017 with the aim of assessing long-term effects of nusinersen in terms of clinical changes and tolerability, and is still ongoing.

#### 2.1.2. Small Molecules

Small molecules that modulate SMN2 gene splicing are currently entering the clinical arena. The most important advantage of these drugs is the oral route of administration. While the intrathecal administration route of nusinersen limits its effect to motoneurons of the central nervous system, the systemic distribution brought about by oral administration allows for effects in other tissues of the body. On the other hand, the disadvantage is the possibility of off-target effects, as these small molecules can theoretically modulate the transcription of other genes. To prevent this risk, a high-throughput in vitro screening was performed to determine molecules highly specific for exon 7 inclusion, and RG7800 and RG7916 were identified.

RG7800. The safety, tolerability, pharmacokinetics, and pharmacodynamics of RG7800 were tested in a multicenter, randomized, double blind, placebo controlled, multiple dose study. This trial was designed with a window of 12 weeks of treatment in adult and pediatric SMA patients (MOONFISH ClinicalTrials.gov identifier: NCT02240355, conducted over the years 2014–2015). After a single dose of the compound, the level of full-length SMN2 mRNA significantly increased in a dose-dependent manner. Unfortunately, long-term (39-weeks) treatment with RG7800 in monkeys showed eye toxicity and for this reason the study was stopped soon after the enrolment of the first group of patients.

RG7916. A phase I study of RG7916 in healthy volunteers (ClinicalTrials.gov identifier: NCT02633709, conducted in 2016) identified the optimal dosage and demonstrated that RG7916 increased SMN2 mRNA levels in humans in a dose-dependent manner. Three ongoing phase II trials are assessing safety and effectiveness of RG7916 in SMA I, II, and III patients. SUNFISH (ClinicalTrials.gov identifier: NCT02908685) is assessing safety, tolerability and effectiveness of RG7916 in SMA II and III cases aged 2–25 years who are not ambulatory; the trial started in 2016 and is still ongoing [10]. Part 1 of SUNFISH evaluates the safety, pharmacodynamics, pharmacokinetics, and optimal dosage of RG7916, while its efficacy is tested versus placebo with a 2:1 randomization in Part 2. FIREFISH (ClinicalTrials.gov identifier: NCT02913482, started in 2016 and still ongoing)—is an open-label trial testing the effectiveness of RG7916 in SMA I patients between one and seven months of age with two copies of SMN2. Part 1 of FIREFISH tests RG7916 in increasing dosage, while Part 2 assesses the effectiveness at the selected dose. JEWELFISH (ClinicalTrials.gov identifier: NCT03032172, started in 2017 and still ongoing) is an open-label, exploratory study that tests the safety and effectiveness of RG7916 in SMA patients who have previously enrolled in trials with SMN2-targeted treatments.

### 2.2. SMN1 Gene Replacement

SMN1 gene replacement therapies using adeno-associated viral (AAV) vectors have demonstrated encouraging effects in in vivo studies and in one phase I clinical trial [11,12]. The main advantage of this approach is that a single intravenous administration would lead to systemic expression of the SMN1 protein, which could ameliorate the multi-organ clinical effects of SMA. Safety data monitoring is crucial, as acute hepatotoxicity and sensory neuron toxicity have been shown in in vivo studies following high systemic administration of AAV vectors [13].

The first AveXis trial (ClinicalTrials.gov identifier: NCT02122952, conducted over the years 2014–2017) was a phase I gene transfer clinical study of 15 SMA I cases with bi-allelic SMN1 mutations (deletion or point mutations) and two copies of SMN2 [11,14]. AVXS-101, which is an AAV9 vector carrying SMN1 complementary recombinant DNA, was administered as a single intravenous dose (12 patients receiving a “high dose” and three receiving a “low dose”) and the clinical course was compared with historical controls from the NeuroNEXT study [15]. Serum aminotransferase levels increased in four cases, but returned to normal levels after treatment with corticosteroids. At 20 months of age, all treated patients were alive and did not need permanent ventilatory assistance, compared to the 8% survival rate in the historical cohort. In the high-dose group, scores on the functional scale Children’s Hospital of Philadelphia Infant Test of Neuromuscular Disorders (CHOP INTEND) increased 9.8 points after one month and 15.4 points after three months, compared to the decrease observed in the historical controls. Of the 12 patients in the high-dose group, 11 patients acquired head control, 11 were able to sit unsupported, nine patients rolled, and two patients gained the ability to walk without support, while none of these motor milestones were reached by the control subjects [11]. Moreover, the majority of patients without ventilatory or nutritional assistance at baseline did not require any intervention during the study. The functional improvements were continuous over the two-year trial period; the results of the ongoing AveXis 302 study (ClinicalTrials.gov identifier: NCT03461289, started in 2018) with long term outcomes and larger cohorts are pending.

## 3. Treatments Targeting Survival Motor Neuron (SMN) Independent Factors

Several clinical trials examined the effects of treatments that focus on non-SMN targets, which can be relevant for patients excluded from SMN-dependent gene therapies or as an “add-on” treatment to supplement genetic therapies.

### 3.1. Neuroprotective Therapies

One of the most promising neuroprotective agents is olesoxime (TRO19622), a novel cholesterol-like compound selected by high-throughput screening aimed at motoneuron survival. Preclinical studies suggested that olesoxime protects mitochondrial activity by decreasing mitochondrial membrane fluidity, and reduces muscle denervation, astrogliosis, microglial activation, and loss of motoneurons [16].

Safety and effectiveness of olesoxime were assessed in a phase II randomized, double-blind, adaptive, placebo-controlled, three-stage study in SMA 2 or non-ambulatory SMA 3 patients (ClinicalTrials.gov identifier: NCT01302600, conducted over the years 2014–2017). Patients were randomly allocated to receive an oral liquid suspension of olesoxime (10 mg/kg per day) or placebo (allocation 2:1) and were followed for 24 months. Although the primary aim of looking for improvement in functional domains 1 and 2 of the Motor Function Measure compared to the placebo group was negative, a significantly higher percentage of treated patients either showed improvement or remained stable (response rate) on the Hammersmith Functional motor scale compared to controls [17]. Answering requests from regulatory authorities for additional data on efficacy and long-term safety, an open-label, single-arm, phase II trial (ClinicalTrials.gov identifier: NCT02628743, started in 2017) of the original subjects is ongoing to assess the safety profile and efficacy of olesoxime over a five-year period.

### 3.2. Muscle Enhancing Therapies

Tirasemtiv (CK-2127107) from Cytokinetics is an activator of troponin in fast skeletal muscles that slows the release of calcium from the regulatory troponin complex, sensitizing the sarcomere to calcium effects and reinforcing contraction [18]. This compound has also been shown to enhance skeletal muscle reaction to nerve impulses in humans [19] leading to the hypothesis that Tirasemtiv might strengthen muscle contraction in SMA patients. Following a phase I study that demonstrated its safety, a phase II, double-blind, randomized, placebo-controlled trial (ClinicalTrials.gov identifier: NCT02644668, conducted over the years 2015–2018) on patients with SMA II to SMA IV, examined the effect of Tirasemtiv on functional performances for eight weeks.

SRK-015 (ScholarRock) is a selective and local inhibitor of latent myostatin that promotes growth and differentiation of muscle cells, and improves muscle force in SMA mice [20]. A phase I trial is ongoing (ClinicalTrials.gov identifier: NCT02644777, started in 2017), and phase II is planned for 2019.

## 4. Discussion

Each therapeutic strategy has its strengths and weaknesses [21,22] and clinicians need to understand them in order to provide care that is suitable for each patient. The results of nusinersen trials raised noticeable enthusiasm within the SMA community, but the intrathecal administration and the related safety requirements (e.g., hospitalization for severe cases) added a significant burden to clinicians as well as to the children receiving treatment and their families. The high financial burdens associated with nusinersen treatment (drug and hospitalization costs) highlight the need to establish the infrastructure and programs necessary to support patients. Furthermore, the efficacy of nusinersen led to a more proactive approach in the management of type I SMA cases, but created frustration in patients with severe scoliosis or spinal instrumentation surgeries because they were excluded from treatment. These ethical, financial, and organizational concerns need to be addressed by hospital administrators, clinicians, and patient support groups so that all SMA patients can be given access to the treatment that they require.

The advantage of the SMN1 gene replacement therapies is that a single administration can lead to continuous functional improvement over two years, as demonstrated in the AVXS-101 trial. The disadvantages stem from the possible safety concerns related to the systemic administration of AAV vectors. The results of the AveXis 302 study are particularly important, as this study will elucidate the efficacy and safety in a larger cohort of patients over a longer period.

Nowadays, it is difficult for experts in this field to select the best treatment strategy. At the moment, only nusinersen is available without limitations in SMA patients, and we think that the different options in terms of clinical trials and available treatments have to be presented to the families and patients. Once other approaches, such as gene therapy, are available for all SMA patients, the final choice will be based on the patient’s clinical status, possible compliance and the feasibility of drug administration. For example, SMA type II patients with severe scoliosis will preferably be treated with gene therapy due to the ease of administration. Once other therapies are available, a treatment flow chart made by a consensus of experts in the field, that outlines the decision-making process, will be required.

Hopefully, the results of the ongoing studies will provide information to aid clinicians in selecting the treatment that is best suited for their patients, with the increasing knowledge on the genetic background of SMA and the evolving treatment strategies, including gene therapies, that are being developed.

## 5. Conclusions

Early diagnosis, including newborn screening, is desirable and access to these new and effective treatments is vital to provide optimal care. Additional therapies to complement present and forthcoming SMN-targeted treatments are needed in order to optimize their effects. During the last few years, scientists and clinicians have worked together towards finding a cure for SMA patients with impressive results. Indeed, nusinersen has been approved for the treatment of all types of SMA, although data on the efficacy in type III and IV SMA are still relatively sparse. Finding therapies that are efficient across SMA clinical phenotypes will be a complex challenge and results, particularly for milder forms of the disease, are likely to take a long time to establish. Furthermore, it is to be expected that future trial design will be complex, as the effect of nusinersen on the clinical progress of SMA is still incompletely understood, and yet many patients are likely to begin treatment soon. Comparing the efficacy of nusinersen to other SMN-targeted therapies will be challenging, as delivery routes can be complex and washout periods can be long. Indeed, the design of future studies investigating the efficacy of combinatorial therapies will be even more challenging as many patients will likely have already received one or more SMN-targeting drugs. Continuing complementary efforts by scientists, clinicians and SMA advocacy groups are therefore required to improve the clinical outcomes of future SMA patients.

## Figures and Tables

**Table 1:** Characteristics of different clinical trials with nusinersen.

	SMA I			SMA II and III	
	Nurture	Endear	CS3A	Cherish	CS12
Trial design	Phase II, open-label, single-arm	Phase III, randomized, double-blind, placebo-controlled	Phase III, open-label, single-arm	Phase III, randomized, double-blind, placebo-controlled	Phase I, open label, single-arm
Patients	Presymptomatic SMA I, 2 or 3 SMN2 copies	SMA I, 2 SMN2 copies	SMA I	SMA II	SMA II and III
Age at screening	≤6 weeks	≤7 months	3 weeks–7 months	2–12 years	2–15 years
Primary endpoints	Time to lethality or respiratory intervention (tracheostomy or ≥6 h ventilator use)	Time to lethality or permanent ventilation, change in Hammersmith Infant Neurologic Exam (HINE-2)	Change in Hammersmith Infant Neurologic Exam (HINE-2)	Change in Hammersmith Functional Motor Scale-Expanded (baseline–15 months)	Safety profile
Length	Started in 2015 (ongoing)	13 months (ended after interim analysis)	45 months	15 months (ended after interim analysis)	45 months
Results	Improvement compared to natural history data for SMA 1	Improvement compared to sham	Improvement compared to natural history data for SMA	Improvement compared to sham	Nusinersen is safe and tolerable
